# Health Behaviors and Experiences of LGBTQ + Individuals during 2022 Mpox Outbreak: Findings from the QVax Study

**DOI:** 10.1007/s10900-024-01383-0

**Published:** 2024-08-25

**Authors:** Kristen D. Krause, Kendra Lewis, Stephan Scrofani, Tiffany Y. Guo, Davin Goulbourne, Perry N. Halkitis

**Affiliations:** 1 Center for Health, Identity, Behavior, and Prevention Studies, School of Public Health (CHIBPS), Rutgers University, One Riverfront Plaza, Suite 1020, Newark, NJ 07102 USA; 2 Department of Urban–Global Health, School of Public Health, Rutgers University, Newark, NJ USA; 3 Department of Biostatistics and Epidemiology, School of Public Health, Rutgers University, Piscataway, NJ USA; 4 Department of Public Health, Montclair State University, Montclair, NJ USA

**Keywords:** Mpox, LGBTQ Health, Vaccinations, Vaccine Uptake

## Abstract

The 2022 global outbreak of Mpox (formerly named Monkeypox) disproportionately impacted lesbian, gay, bisexual, transgender, and queer (LGBTQ+) populations, with a significant impact on sexual minority men. A cross-sectional survey was conducted among LGBTQ + adults living in New Jersey and New York to examine vaccination behaviors for Mpox. We sought out to understand the health experiences and behaviors of the population during the initial outbreak. This analysis included a subset of participants (*n* = 253) who completed the survey after the onset of the Mpox outbreak in May 2022. We examined awareness of and concerns about Mpox, willingness to vaccinate, as well as vaccination access and vaccination uptake. Our findings indicate that white, cisgender, gay men, as well as employed individuals, had the highest rates of vaccination for Mpox. Those with higher levels of concern about the virus were both more likely to be vaccinated and to report difficulty securing an appointment. Overall, our findings reinforce the health disparities in the population, demonstrating as with many other health conditions that white and economically stable individuals have advantages in accessing care.

## Introduction

On May 6, 2022, the first cases of the 2022 Mpox (formerly named Monkeypox) outbreak were reported in the United Kingdom [1]. Soon thereafter, cases were also reported in other non-endemic countries around the world including Portugal and Spain [2–4] which led to the World Health Organization to declare a public health emergency on July 23, 2022 [5]. This outbreaks occurred at a time when COVID-19 was still a public health emergency in the United States (US) [6]. Public health systems worldwide were already burdened by the COVID-19 pandemic, leading many countries to delay their Mpox mitigation strategies. As of March 2023, just over 30,000 cases [7] have been confirmed or deemed probable in the US [7], with cisgender men who have sex with other men (MSM) being the most disproportionally impacted population [7, 8].

The relaxation of COVID-19 prevention measures was more prevalent during 2022 which allowed the public to resume various activities including travel and sexual interactions that have been associated with large gatherings [9]. As COVID-19 cases began decreasing and public health emergencies associated with it were lifted, Mpox saw a sudden rise, placing the infectious disease at the forefront of public health priorities during the summer of 2022 [10]. Mpox is primarily transmitted through sexual contact [11], which is a main contributing factor explaining why gay men, bisexual, and other men who have sex with men (sexual minority men, SMM) have been disproportionately impacted [12]. By November 2022, roughly 93 − 98% of reported Mpox cases were detected in SMM as well as heterosexually identified MSM [13] Between May and November 2022, 466 cases were reported among transgender and gender-diverse adults [11].

Vaccinations for Mpox exist and can be given before or after exposure to the virus as a preventative measure or as a way to reduce symptoms [2]. Because Mpox is in the same family as the smallpox virus, the smallpox vaccine was utilized (JYNNEOS) [2]. For those who could not receive the JYNNEOS vaccine because of immunosuppression, an alternative vaccine, ACCAM, was utilized [2]. The JYNNEOS vaccination follows a two dose schedule 28 days apart, while the ACAM2000 vaccination can be delivered in one dose, but requires a more difficult injection method [2]. Despite there being an effective vaccine, the US did not have enough JYNNEOS vaccines in reserve to ensure that all members of populations who were at risk could receive both doses of the JYNNEOS vaccination, and a worldwide shortage further complicated the rollout [14]. To ensure that the target population still received the vaccine, guidelines changed from two subcutaneous doses to one subcutaneous dose and two intradermal doses. Across the US, Mpox vaccination models and frameworks were created to ensure that LGBTQ+, particularly SMM populations were able to be vaccinated [15]. Tellingly, there are no clinical trial studies to establish the efficacy of the JYNNEOS vaccination for Mpox [16], but the CDC reported that those SMM not vaccinated were seven times more likely to test positive for Mpox than those in the same population that had at least one dosage [16]. While racial and ethnic disparities existed for Mpox vaccine uptake [17], by the end of 2022, Black men were getting vaccinated at a higher rate than white men for Mpox, however, this higher vaccination rate did not offset the disproportionate number that men of color were testing positive for Mpox [17].

During the COVID-19 pandemic, vaccine resistance was prevalent in many different subpopulations [18]. Our own work has demonstrated much more limited hesitancy in sexual and gender minority (SGM) populations [19, 20]. Compared to the larger US population, people who identify as a SGM are reported to have at least one dose of the COVID-19 vaccination [21]. One study reported that SGM participants felt like getting the vaccination was a way to protect their communities [22]. They also reported that the SGM population were more willing to be vaccinated in general [22].

These analyses examined Mpox vaccination attitudes and behaviors in SGM (i.e. LGBTQ+). We analyzed the relationship between sociodemographic characteristics, Mpox vaccination knowledge and concern, vaccine uptake/intent to vaccinate, and appointment scheduling ease.

## Methods

Between October 2021 and November 2022, we undertook a cross-sectional online survey, referred to as Qvax within the local community. Eligibility criteria included being 18 years or older, self-identifying as LGBTQ+, and residing in either New Jersey (NJ) or New York (NY). All study protocols received approval from the Rutgers University Institutional Review Board (IRB). We utilized Qualtrics XM [23] to host the survey. Recruitment materials with an anonymous link to the survey were distributed via social media, listservs of professional organizations, and NJ- and NY-based LGBTQ + community centers and events to recruit participants. After screening, participants who were eligible provided informed consent and then were directed to the survey. Upon survey completion, participants had an opportunity to enter a raffle to win one of fifteen $30 electronic gift cards.

The Mpox outbreak was evolving at the time of the study. As a result, additional questions about Mpox were added at two time points. In May 2022, prior to the availability of vaccines, we added two items on Mpox awareness and on Mpox concern (e.g. phase 2). In July of 2023, once the vaccine became available in the US, we added additional questions about uptake and access of the vaccination (e.g. phase 3).

Based on previous experiences with bots and “bad actors” we undertook data cleaning in a method developed by our team [24]. Records were removed if they met any of the following criteria: completed less than 75% of the entire survey, submitted duplicate answers for qualitative questions, did not provide a valid zip code, and received a Google reCAPTCHA score [25] below 0.5. We also removed records where the county of residence and zip code did not align; however, special considerations were made if the zip code was geographically (e.g., Erie and Niagara) or alphabetically (e.g., Hudson and Hunterdon) adjacent. All questionable records were cross-checked by two study team members. The overall study sample included *n* = 768 participants; phase 2 included a subset of *n* = 253 who answered all study questions and the newly added Mpox questions; and phase 3 included a smaller subset of *n* = 144. The three samples are shown in Table 1 and demonstrate comparability in terms of characteristics.

**Table 1:** Sociodemographic and Health characteristics of study samples

	Full Sample	Phase 2 Sample (May 2022-October 2022)	Phase 3 Sample (July 22, 2022-October 2022)
	*N* = 768 % (n)	*N* = 253 % (n)	*N* = 144 % (n)
Age			
18–29	46.0 (353)	53.4 (135)	50.0 (72)
30–39	30.5 (234)	28.5 (72)	25.7 (37)
40–49	8.5 (65)	7.9 (20)	11.1 (16)
50 +	15.1 (116)	10.3 (26)	13.2 (19)
Race/ethnicity			
American Indian, non-Hispanic	0.8 (6)	-	0.7 (1)
Asian, non-Hispanic	5.6 (43)	9.1 (23)	9.7 (14)
Black, non-Hispanic	7.6 (58)	10.7 (27)	12.5 (18)
Hispanic/Latino	11.7 (90)	9.9 (25)	9.0 (13)
Middle Eastern, non-Hispanic	0.4 (3)	-	-
Native Hawaiian, non-Hispanic	0.5 (4)	-	0.7 (1)
White, non-Hispanic	68.2 (524)	63.2 (160)	61.8 (89)
Other, non-Hispanic	0.5 (4)	-	-
Mixed, non-Hispanic	4.7 (36)	-	5.6 (8)
Gender w/ Non-binary/ Other			
Cisgender Man	28.9 (222)	28.5 (72)	30.6 (44)
Cisgender Woman	33.7 (259)	34.8 (88)	34.0 (49)
Transgender Man	8.7 (67)	5.9 (15)	6.9 (10)
Transgender Woman	5.7 (44)	7.1 (18)	4.9 (7)
Nonbinary/Different Gender Identity	22.9 (176)	23.7 (60)	23.6 (34)
Sexual Orientation			
Asexual/Queer/Other	21.7 (167)	22.5 (57)	22.9 (33)
Bisexual/Pansexual	25.0 (192)	25.3 (64)	27.8 (40)
Gay	33.6 (258)	30.0 (76)	28.5 (41)
Lesbian	18.0 (138)	22.1 (56)	20.8 (30)
Heterosexual or Straight	1.7 (13)	-	-
Employment			
Full Time	62.9 (483)	58.1 (147)	52.8 (76)
Part Time	20.2 (155)	22.1 (56)	22.9 (33)
Unemployed	16.5 (127)	19.4 (49)	24.3 (35)
HIV Status			
HIV Negative	85.3 (655)	82.2 (208)	86.8 (125)
HIV Positive	6.5 (50)	7.1 (18)	6.9 (10)
HIV Status Unknown	8.1 (62)	10.7 (27)	6.3 (9)
Health Insurance			
Yes, Public or Private	95.7 (735)	93.7 (237)	94.4 (136)
No	4.2 (32)	5.9 (15)	4.9 (7)
Nation of Birth			
United States	91.3 (701)	92.7 (228)	87.5 (126)
Other	7.4 (57)	7.3 (18)	7.6 (11)
State of Residence			
New Jersey	73.0 (561)	54.5 (138)	56.3 (81)
New York	27.0 (207)	45.5 (115)	43.8 (63)

### Measures

All 768 participants completed the following measures.

### Demographics

Participants provided self-reported data on a myriad demographics. Age was re-coded into four categories (18–29, 30–39, 40–49, and ≥ 50). Participants also reported their sex assigned at birth (male/female), gender identity (male, female, transgender man, transgender woman, non-binary/genderqueer/gender non-conforming, or an open text option), and sexual orientation (gay, lesbian, bisexual, asexual, queer, heterosexual/straight, or an open text option). For race and ethnicity, participants were asked to choose all that apply: Hispanic/Latino, American Indian/Alaska Native, Black/African American, Asian, Middle Eastern or North African, Native Hawaiian or Pacific Islander, White, or “other”. Responses were used to create the following analytic groups: Hispanic/Latino, Black non-Hispanic, Asian/Native Hawaiian or Pacific Islander non-Hispanic, American Indian/Alaska Native/ Middle Eastern or North African/other non-Hispanic, multiracial non-Hispanic, and White non-Hispanic.

Initially, for a more nuanced understanding around the intersectionality of sexual orientation and gender identity (SOGI), we categorized participants into 15 groups for each combination of SOGI intersections. Due to small cell sizes among these categories, gender identity and sexual orientation were coded as separate variables for analysis. For gender identity, the categories included cisgender man, cisgender women, transgender man, transgender woman, and non-binary/different gender identity. For sexual orientation, the categories included gay, lesbian, heterosexual/straight, bi-sexual/pansexual, and asexual/queer/other.

We also gathered data on other sociodemographic variables, including employment (unemployed, employed full-time/part-time), nation of birth (US, outside US), and state of residence (NJ, NY). Additionally, participants self-reported their HIV status (positive, negative, unknown).

### Vaccination for Other Vaccine Preventable Diseases (VPD)

We asked participants to report history of vaccination for other sexually transmitted VPD including Human Papilloma Virus (HPV), Hepatitis A (Hep A), Hepatitis B (Hep B), and Meningitis B (Men B). We also asked participants about their thoughts on a future HIV vaccination. Vaccination for other VPD’s are not presented in the current study.

The following Mpox Items were added in May 2022 (phase 2).

### Mpox Awareness and Concerns

After it was clear that the reemergence of Mpox in May 2022 was predominately affecting members of LGBTQ + populations, we added two questions to our survey asking whether participants had heard about the recent Mpox outbreak (yes/no) and if they said yes (*n* = 225), we asked about their level of concern about contracting Mpox using a 5-point Likert scale (1 = not at all concerned to 5 = extremely concerned). For analysis we re-coded Mpox concern into three groups including as (1) not/slightly concerned, (2) somewhat concerned, and (3) moderately/very concerned (*n* = 224).

The following Mpox Items were added in July 2022 (phase 3).

### Mpox Uptake, Difficulty Securing an Appointment, and Availability

After a vaccine became more widely available to sexual minority men and gender minority individuals, on July 22, 2022, we added three additional questions for those who had heard about Mpox (*n* = 144). First, we assessed whether participants had received the Mpox vaccine (yes, I have received one dose; yes, I have received two doses; no, but I intend to; and no, I do not intend to). For analysis, we reclassified participants based on intent: (1) yes to either dosage or no vaccination, but I intend to, and (2) not vaccinated and I do not intend to.

Among participants who indicated “yes” or “ I intend to be vaccinated”, we assessed their difficulty scheduling an appointment using a 5-point Likert scale (1 = not at all difficult to 5 = very difficult). Later for analysis, difficulty securing an appointment was re-coded into two groups, including: (1) Not/ Slightly/Somewhat Difficult, and (2) Moderately/ Very difficult. Finally, among participants who had heard about Mpox, we asked about their likelihood of getting the vaccine, provided that availability is easy using a 5-point Likert scale (1 = very unlikely to 5 = very likely). For analysis, groups were re-coded into three categories, 1 = very unlikely or unlikely, 2 = neither likely or unlikely, and 3 = likely or very likely.

### Statistical Analysis

Descriptive statistics were computed for all sociodemographic characteristics, Mpox items, and vaccination attitudes and uptake. We then analyzed the relationship between the Mpox items and sociodemographic characteristics. We also examined bivariate relationships among the Mpox items themselves, and between Mpox items, vaccination attitudes, and Mpox uptake. Due to limited sample size for the Mpox items, many bivariate analyses were conducted using likelihood ratio testing rather than the more traditional non-parametric approach, chi-square χ^2^ tests of independence. Due to small cell sizes, chi-square test of independence was applied only if at least 75% of expected count for cells were greater than 5. All analyses were conducted with SPSS V28.

## Results

Our total sample included 768 adults who were eligible and deemed to be actual participants. Of this sample, 253 participants completed the survey with two added Mpox items after May 22, 2022 (phase 2). Three more questions were added after July 22, 2022, and 144 participants completed questions after this date (phase 3). In the phase 2 sample (*n* = 253) the majority of the participants (53.4%) were between the ages of 18 and 29 (Table 1). Most of the sample included White, non-Hispanic participants (63.2%), employed full-time (58.1%), who identify as gay (30%). Cisgender women (34.8%) and cisgender men (28.5%) made up the largest proportion of participants, followed by non-binary/different gender identity (23.7%) identified participants. However, less participants identified as transgender women (7.1%) and transgender men (5.9%). Overall, the majority of participants reported that they have either private or public health insurance (93.7%), and that they reside in New Jersey (54.5%). Additional sociodemographic information for the phase 2 sample is shown in Table 1.

The sub-sample of participants retained after July 22, 2022 (phase 3) have similar sociodemographic characteristics to the May 22, 2022 sub-sample (phase 2). More frequency data on sociodemographic information for phase 3 is shown in Table 1, alongside full, and phase 2 sample statistics.

Table 2 shows side-by-side comparisons of frequencies of the full sample, phase 2, and phase 3 sub-samples on key variables pertaining to Mpox thoughts and behavior. In phase 2, the majority of participants (90.0%) had reported that they had heard of the recent Mpox outbreak. Related, most participants reported that they were concerned about contracting Mpox (57.6%), including 24.6% that were somewhat concerned, and 33% that were either moderately or very concerned. Similar to the phase 2 sample, all (100%) of participants in the July 22, 2022 sub-sample (phase 3) had reported that they had heard of the recent Mpox outbreak. Also, among participants from phase 3, most participants reported that they were concerned about contracting Mpox (68.1%), including 25% that were somewhat concerned, and 43.1% that were either moderately or very concerned.

As of July 22, 2022 (phase 3), 73.6% (*n* = 106) of participants reported that they either had received one or two doses of the Mpox vaccine, or that they intend to (Table 2). About half (51.5%; *n* = 51) of participants who answered the Mpox questions after July 22, 2022 (phase 3) said it was either moderately or very difficult to make an appointment for the Mpox vaccination (Table 2). Similarly, 67.5% (*n* = 54) reported that they would be either likely or very likely to take the Mpox vaccination, if it was easily available (Table 2).

Among the phase 3 sample (*n* = 144), we assessed the bivariate relationship between key demographics with Mpox uptake (vaccination status), difficulty securing an appointment, and intent to vaccinate, provided that access is easy (Table 3). Those who had not heard about Mpox were not included in the ensuing analyses about vaccination. First, nonparametric tests of independence between sociodemographic characteristics and Mpox vaccination status were ran. When participants were asked if they had received at least one dose of the Mpox vaccine or if they intend to, cisgender men (90.9%; (χ^2^ (4) = 13.22, *p* = .010), those identified as gay (95.1%; (χ^2^ (3) = 15.74, *p* = .001) and full-time employees (81.6%; χ^2^ (2) = 5.82, *p* = .055) were more likely to receive at least one dose of the Mpox vaccine or intend to be vaccinated, compared to other groups in the phase 3 sample.

**Table 2:** Frequency statistics for Mpox thoughts and behaviors

	Full Sample	Mpox Sub-sample	July 22nd
	*N* = 768 % (n)	*N* = 253 % (n)	*N* = 144
Have you heard of the recent Mpox outbreak?			
Yes	-	90.0 (225)	100.0 (144)
No	-	10.0 (25)	0.0 (0)
How concerned are you about contracting Monkeypox because of the recent outbreak?			
Not/ Slightly concerned	-	42.4 (95)	31.9 (46)
Somewhat concerned	-	24.6 (55)	25.0 (36)
Moderately/ Very concerned	-	33.0 (74)	43.1 (62)
As of July 22, 2022, have you received the Monkeypox vaccine?			
Yes, I have received one/two doses OR No, but I intend to	-	-	73.6 (106)
No, and I do not intend to	-		26.4 (38)
How difficult has it been (or was it) to secure an appointment to receive a Monkeypox vaccine?		-	
Not/Slightly/Somewhat Difficult	-	-	48.5 (48)
Moderately/Very Difficult	-	-	51.5 (51)
If the monkeypox vaccine became easily available to you, how likely would it be that you would take the vaccine?			
Very Unlikely/Unlikely	-	-	12.5 (10)
Neither Likely or Unlikely	-	-	20.0 (16)
Likely/Very Likely	-	-	67.5 (54)

**Table 3:** Correlates of Mpox Vaccine Uptake and Access among participants in a Regional Survey of LGBTQ +people NJ and NY, 2022

	MPOX Vaccination *n* = 144			Difficulty Securing an Appointment for MPOX Vaccination *N* = 99			Likelihood of MPOX Vaccination with Easy Availability *N* = 80			
	Yes, I have received one/two doses/No, but I intend to % (*n*) [AR]	No, and I do not intend to % (*n*) [AR]	*p*-value	Not/ Slightly/ Somewhat Difficult % (*n*) [AR]	Moderately/ Very Difficult % (*n*) [AR]	*p*-value	Very Unlikely/ Unlikely % (*n*) [AR]	Neither Likely nor Unlikely % (*n*) [AR]	Likely/Very Likely % (*n*) [AR]	*p*-value
Age Group			0.982^			0.185				0.285^
18–29	72.2 (52)	27.8 (20)		51.0 (25)	49.0 (24)		15.6 (7)	15.6 (7)	68.9 (31)	
30–39	75.7 (28)	24.3 (9)		46.2 (12)	53.8 (14)		12.0 (3)	32.0 (8)	56.0 (14)	
40–49	75.0 (12)	25.0 (4)		20.0 (2)	80.0 (8)		0.0 (0)	0.0 (0)	100.0 (3)	
50+	73.7 (14)	26.3 (5)		64.3 (9)	35.7 (5)		0.0 (0)	14.3 (1)	85.7 (6)	
Race/Ethnicity			0.309^			0.320^				0.181^
White, non-Hispanic	68.5 (61)	31.5 (28)		41.8 (23)	58.2 (32)		15.1 (8)	20.8 (11)	64.2 (34)	
American Indian/MEA/Other, non-Hispanic	100.0 (1)	0.0 (0)		100.0 (1)	0.0 (0)		0.0 (0)	50.0 (1)	50.0 (1)	
Asian/Native Hawaiian, non-Hispanic	86.7 (13)	13.3 (2)		53.8 (7)	46.2 (6)		0.0 (0)	0.0 (0)	100.0 (8)	
Black, non-Hispanic	88.9 (16)	11.1 (2)		46.7 (7)	53.3 (8)		25.0 (1)	25.0 (1)	50.0 (2)	
Hispanic/Latino	69.2 (9)	30.8 (4)		77.8 (7)	22.2 (2)		0.0 (0)	37.5 (3)	62.5 (5)	
Multicultural, non-Hispanic	75.0 (6)	25.0 (2)		50.0 (3)	50.0 (3)		20.0 (1)	0.0 (0)	80.0(4)	
Gender Identity			**0.010**			0.113^				0.478^
Cisgender Man	90.9 (40) [3.1]	9.1 (4) [-3.1]		45.0 (18)	55.0 (22)		4.2 (1)	16.7 (4)	79.2 (19)	
Cisgender Woman	59.2 (29) [-2.8]	40.8 (20) [2.8]		42.3 (11)	57.7 (15)		21.4 (6)	21.4 (6)	57.1 (16)	
Transgender Man	80.0 (8) [0.5]	20.0 (2) [-0.5]		87.5 (7)	12.5 (1)		0.0 (0)	33.3 (1)	66.7 (2)	
Transgender Woman	57.1 (4) [-1.0]	42.9 (3) [1.0]		75.0 (3)	25.0 (1)		33.3 (1)	0.0 (0)	66.7 (2)	
Non-binary/Different Identity	73.5 (25) [ 0.0]	26.5 (9) [0.0]		42.9 (9)	57.1 (12)		9.1 (2)	22.7 (5)	68.2 (15)	
Sexual Orientation			**0.001**			0.627				**0.035^**
Asexual/Queer/Other	72.7 (24) [-0.1]	27.3 (9) [0.1]		34.8 (8)	65.2 (15)		17.6 (3) [0.7]	29.4 (5) [1.1]	52.9 (9) [-1.4]	
Bisexual/Pansexual	65.0 (26) [-1.5]	35.0 (14) [1.5]		52.2 (12)	47.8 (11)		10.5 (2) [-0.3]	5.3 (1) [-1.8]	84.2 (16) [1.8]	
Gay	95.1 (39) [3.7]	4.9 (2) [-3.7]		53.8 (21)	46.2 (18)		3.4 (1) [-1.8]	17.2 (5) [-0.5]	79.3 (23) [1.7]	
Lesbian	56.7 (17) [-2.4]	43.3 (13) [2.4]		50.0 (7)	50.0 (7)		26.7 (4) [1.8]	33.3 (5) [1.4]	40.0 (6) [-2.5]	
Employment Status			**0.055**			0.068				0.466^
Yes, full-time employment	81.6 (62) [2.3]	18.4 (14) [-2.3]		42.6 (26)	57.4 (35)		15.7 (8)	17.6 (9)	66.7 (34)	
Yes, part-time employment	60.6 (20) -[1.9]	39.4 (13) [1.9]		40.0 (6)	60.0 (9)		5.3 (1)	31.6 (6)	63.2 (12)	
No, I am unemployed	68.6 (24) [-0.8]	31.4 (11) [0.8]		69.6 (16)	30.4 (7)		10.0 (1)	10.0 (1)	80.0 (8)	
HIV Status			0.439			**0.011^**				0.073
HIV Negative	72.8 (91)	27.2 (34)		45.2 (38) [-1.5]	54.8 (46) [1.5]		12.5 (8)	14.1 (9)	73.4 (47)	
HIV Positive	90.0 (9)	10.0 (1)		44.4 (4) [-0.3]	55.6 (5) [0.3]		0.0 (0)	40.0 (2)	60.0 (3)	
HIV Status Unknown	66.7 (6)	33.3 (3)		100.0 (6) [2.6]	0.0 (0) [-2.6]		18.2 (2)	45.5 (5)	36.4 (4)	
			0.903^							
Health Insurance	73.5 (100)	26.5 (36)				**0.006^**				0.819^
Yes, public or private	71.4 (5)	28.6 (2)		46.2 (43) [-2.3]	53.8 (50) [2.3]		12.3 (9)	19.2 (14)	68.5 (50)	
No			0.938^	100.0 (5) [2.3]	0.0 (0) [-2.3]		14.3 (1)	28.6 (2)	57.1 (4)	
Born in the United States	73.8 (93)	26.2 (33)				0.496^				0.495^
Yes	72.7 (8)	27.3 (3)		50.0 (43)	50.0 (43)		13.3 (10)	20.0 (15)	66.7 (50)	
No			0.536	62.5 (5)	37.5 (3)		0.0 (0)	20.0 (1)	80.0 (4)	
State of Residence	71.6 (58)	28.4 (23)				0.633				0.190
New Jersey	76.2 (48)	23.8 (15)		46.3 (25)	53.7 (29)		14.9 (7)	25.5 (12)	29.6 (28)	
New York				51.1 (23)	48.9 (22)		9.1 (3)	12.1 (4)	78.8 (26)	

^indicates likelihood ratio value reported instead of Chi-square due to sample size limitations

Next, nonparametric tests of independence were run between sociodemographic characteristics and phase 3 questions added (July 22, 2022) that probe about access to Mpox vaccinations (Table 3). Participants that reported HIV status unknown were more likely to report no or low difficulty securing an appointment for Mpox vaccination (*p* = .011). However, the observed frequency is very low among participants that reported unknown HIV status (6). Adjusted residuals (with values approaching the 1.96 +/- threshold) suggest that participants with HIV negative status were more likely to report moderate to strong difficulty securing an appointment for Mpox vaccination (AR = 1.5). In other words, this combination also contributes to the significance of the relationship observed. At the same time, the negative adjusted residual for the cell aligned with HIV negative participants who reported no or low difficulty securing an appointment suggests that less participants were observed here than what the test expected (AR = -1.5).

Contrary to what we anticipated, participants with health insurance (private or public) reported difficultly securing an appointment for a Mpox vaccination (*p* = .006). However, it is unclear if participants without health insurance contend with challenges to secure an appointment since a very small proportion of individuals reported that they did not have insurance. The observed frequencies show that among those with private or public health insurance, 50 reported that it was moderately or very difficult to secure an appointment for a Mpox vaccine (AR = 2.3), while 43 did not (AR = -2.3). Conversely, among those without health insurance, 0 reported moderate or a lot of difficulty (AR = -2.3), and 5 did not (AR = 2.3).

Also pertaining to access, we asked participants about the likelihood that they would vaccinate, if it was easily available. Among these findings a significant association was found between sexual orientation and interest in vaccinating for Mpox, should it become easily accessible (*p* = .035). Although cell sizes are small across four categories for sexuality, findings suggest that lesbians, gay men, and bisexual/pansexual are more likely to report stronger intentions to vaccinate, should the Mpox vaccine become easily accessible. While the combination between asexual/queer/other identified participants and interest in vaccinating at an accessible location is not contributing to the significant relationship.

Next, we examined bivariate relationships among Mpox items from phase 2 and phase 3 (Table 4). Among these relationships we tested the association between concern about contracting Mpox (phase 2) and access to Mpox vaccinations (phase 3) to examine how this individual level factor impacts the ability to vaccinate (Table 4). Chi-square test of independence demonstrated that participants that received 1–2 doses of Mpox vaccine, or intend to, are more likely to report moderate to a lot of concern about contracting Mpox due to the recent outbreak (χ^2^ (2) = 52.71, *p* < .001). Related, chi-square test showed that participants that reported moderate or a lot of difficulty securing an appointment are more likely to report moderate or a lot of concern about contracting Mpox (χ^2^ (2) = 10.13, *p* = .006). See Table 4.

**Table 4:** Mpox Uptake and Access by Mpox concern among participants in a Regional Survey of LGBTQ + people NJ and NY, 2022

	Concerned About Contracting MPOX *n* = 224			
	Not/ Slightly % (*n*) [AR]	Somewhat % (*n*) [AR]	Moderately/ Very % (*n*) [ AR]	*p*-value
**Received the MPOX Vaccination**				**< 0.001**
Yes, I have received 1–2 doses, or I intend to	15.1 (16) [-7.2]	30.2 (32) [2.4]	54.7 (58) [4.7]	
No, and I don’t intend to	78.9 (30) [7.2]	10.5 (4) [-2.4]	10.5 (4) [-4.7]	
**Likelihood of MPOX Vaccination with Easy Availability**				0.192^
Very Unlikely/Unlikely	90.0 (9)	10.0 (1)	0.0 (0)	
Neither Likely or Unlikely	62.5 (10)	25.0 (4)	12.5 (2)	
Likely/Very Likely	55.6 (30)	25.9 (14)	18.5 (10)	
**Difficult Securing an Appointment for a Mpox Vaccination**				**0.006**
Not/Slightly/Somewhat Difficult	22.9 (11) [2.1]	37.5 (18) [1.7]	39.6 (19) [-3.1]	
Moderately/Very Difficult	7.80 (4) [-2.1]	21.6 (11) [-1.7]	70.6 (36) [3.1]	

## Discussion

To review, we assessed the relationship between sociodemographic characteristics and Mpox items (i.e., general knowledge, concern, vaccine uptake/intent, and appointment scheduling ease) in a sample of LGBTQ + individuals residing in NJ and NY. The current study helps to fill the gap in the literature on SOGI data on the overlap of Mpox uptake, access, concern. Despite strain from COVID-19, many local distribution centers pivoted from COVID-19 vaccination and education centers to Mpox vaccination centers, including creating mobile vaccination sites at LGBTQ + events [26]. Still, there was a shortage of vaccinations, which prevented LGBTQ + populations at risk from receiving both doses of the JYNNEOS vaccination [14].

Similar to prior research, vaccination status (which included the intention to be vaccinated for Mpox) showed to be related to gender identity, sexual orientation, and employment status. Especially without full-time employment, monetary challenges can prevent someone from prioritizing Mpox vaccinations at a high cost. Future research that samples black gay males (including cisgender and transgender identities) is needed to examine the intersection of race and sexuality around healthcare access to better understand how these overlapping systems can create challenges to being vaccinated for Mpox. While the current study shows that participants either identified as “gay” or “cisgender male” were more likely to have received the Mpox vaccination or report intention to do so, there is not enough data to assess racial disparities among these participants.

Results suggest that participants who are HIV-negative were more likely to report moderate or more difficulty securing an appointment for an Mpox vaccine. Although cell sizes were small, results demonstrate that participants that do not know their HIV status were more likely to report less or no difficultly securing an appointment for a Mpox vaccine. Historically, these findings allude to how HIV prevention and treatment are communicated in LGBTQ + communities, especially gay males in communities who advocate for healthcare that benefits the community at large. Participants who know their HIV status may be more connected to others in the LGBTQ + community and can therefore encourage others to be tested for HIV consistently [27]. Whereas participants who don’t know their HIV status may be reporting less difficulty because they have not accessed a Mpox vaccination in the first place. Opposing our findings, strict guidelines in the US about who is able to get vaccinated for Mpox can create challenges for those who are HIV-positive or taking medication to prevent HIV [28]. Going forward, a larger proportion of HIV positive participants in the sample can help to untangle why our results contradict prior research that shows that those with HIV positive status have more difficulty accessing a Mpox vaccination. It is possible that people who do not know their HIV status may also not have as much knowledge of Mpox vaccination guidelines or where to receive care in the first place.

Further related to our findings, research based in China shows that people living with HIV (PLWH) had less vaccine hesitation for the Mpox vaccination compared to people who were not living with HIV [29]. Unlike those outside of the LGBTQ + spectrum, traditionally LGBTQ + identities prioritize creating close communities beyond the family network because of strain in family relationships. Prioritizing communities outside of the household for healthcare support could be a motivating force for keeping a vaccination schedule. Beyond this, these findings can be attributed to an urgency for those that are immunocompromised to protect themselves from further health complications.

Additionally, results suggest that gay, lesbian, and bisexual/pansexual participants are more likely than asexual, queer, or “other” identified participants to report that they are likely or very likely to vaccinate for Mpox, provided that it is easy to obtain. Research conducted in Illinois a similar population of LGBTQ + individuals found those identified as gay are more likely to vaccinate for Mpox [30]. Unlike our findings that show that lesbians also are more likely to report intent to vaccinate, provided easy availability, lesbians did not show the same advantage as those identified as gay [30]. These results shed more light on a general interest to vaccinate among gay men, and highlight the need for further research on sexual and gender minority populations that identify outside of gay, bisexual, or pansexual spectrums, and their Mpox vaccination frequencies.

MSM are at a higher risk of testing positive for many sexually transmitted illnesses that could be prevented by vaccinations [31]. The availability of vaccination is reflected in the increased health burden that MSM face if exposed to infection. Uptake for STI vaccinations have been successful with proper interventions tailored to MSM, but there is still a gap in research documenting these interventions, STI status, and vaccination status among MSM and gay men [31]. Many MSM may not be aware that they are susceptible to these diseases. Research shows that when providers talked to their patients who are MSM about the HPV vaccination, they were more likely to be vaccinated [32]. Provider communication that focuses on preventive care for STIs like Mpox is essential in clinical and community health settings.

### Limitations

Our study has a few limitations to note. The limited sample size in participants that completed the Mpox portion of our survey after May 22, 2022, yielded some analytic drawbacks. Where appropriate, we utilized the likelihood ratio test over the chi-square test as many of our analyses had 25% or more cells with less than 5 counts in each. The likelihood ratio test is more robust and accurate when dealing with sparse data, as it employs maximum likelihood estimation to calculate test statistics, resulting in improved statistical power and validity of inference [33].

Some key demographic characteristics of the sample drawn are discrepant from the population at risk. For example, the majority of the sample was made up of white, cisgender males, while Mpox disproportionately affects black, cisgender males. This limitation impacts the extent to which our results on uptake and access can be generalized to the population, which is most at risk for Mpox, black cisgender males. Even though there were three phases of the study, participant demographics in all subsamples are comparable, yielding similar drawbacks in each phase. For all three phases of the study the method for recruiting participants was a convenience sample, so all samples were subject to self-selection bias.

### Future Directions

Going forward, more data is needed on Mpox uptake and sentiments which allows for a nuanced examination of SOGI data. In the current study, a small sample size limited the amount of SOGI categories retained for analysis, rendering some important subgroups invisible (i.e. gay transgender men). Overall, in the literature this gap is most profound among BIPOC and transgender identities and creates challenges measuring the intersectionality around race and sexuality for Mpox vaccination uptake. Given the disproportionate rate that African American cisgender males test positive for Mpox [17] more research sampling BIPOC LGBTQ + can help to unravel how these overlapping systems create strains and supports which contribute to vaccination hesitancy and overall access.

Additionally, there is a need for more data on prosocial behavior to more closely examine the association between attitudes such as community altruism and Mpox vaccination uptake. Although the current study asked participants about their overall concern about contracting Mpox, questions that probe further about concern for self, and concern for others can better unpack the extent to which people are motivated to get vaccinated as a way to protect their community. Furthermore, data on community altruism is valuable going forward because it can help preventive efforts targeting LGBTQ + to create supports to build connectedness in communities.
