# Correction to: Health Behaviors and Experiences of LGBTQ + Individuals during 2022 Mpox Outbreak: Findings from the QVax Study

**DOI:** 10.1007/s10900-024-01420-y

**Published:** 2024-11-22

**Authors:** Kristen D. Krause, Kendra Lewis, Stephan Scrofani, Tiffany Y. Guo, Davin Goulbourne, Perry N. Halkitis

**Affiliations:** 1 Center for Health, Identity, Behavior, and Prevention Studies, School of Public Health (CHIBPS), Rutgers University, One Riverfront Plaza, Suite 1020, Newark, NJ 07102 USA; 2 Department of Urban-Global Health, School of Public Health, Rutgers University, Newark, NJ USA; 3 Department of Biostatistics and Epidemiology, School of Public Health, Rutgers University, Piscataway, NJ USA; 4 Department of Public Health, Montclair State University, Montclair, NJ USA

**Correction to: Journal of Community Health** 10.1007/s10900-024-01383-0

During typesetting process, *χ*^2^ symbols were unfortunately converted incorrectly into Φ2. All of the *χ*^2^ symbols should be Ф2 (chi-square).

In addition, Table 3 should be titled Correlates of Mpox Vaccine Uptake and Access among participants in a Regional Survey of LGBTQ + people NJ and NY, 2022.

The original article has been corrected.

